# Successful surgical treatment for a thrombus straddling a patent foramen ovale: a case report

**DOI:** 10.1186/1749-8090-8-138

**Published:** 2013-05-30

**Authors:** Atsushi Nemoto, Mikihiko Kudo, Kentaro Yamabe, Ryohei Yozu

**Affiliations:** 1 Department of Cardiovascular Surgery, School of Medicine, Keio University, Shinanomachi 35, Shinjuku, Tokyo 160-8582, Japan

**Keywords:** Paradoxical embolism, Patent foramen ovale, Economy class syndrome, Pulmonary embolism

## Abstract

Paradoxical embolism (PDE) occurs after embolic material passes from the venous to the arterial circulation through a right-to-left shunt, which is frequently a patent foramen ovale (PFO). We describe the case of a patient with deep venous thrombosis and an intracardiac thrombus straddling a PFO and who was successfully treated with an emergency surgery.

## Background

“Economy class syndrome” is a well-known condition characterized by a venous thromboembolism associated with long periods of travel [1]. The association between long-distance travel and stroke has been reported as “economy class stroke syndrome,” which typically results from PDE through a PFO and is often accompanied by deep venous thrombosis (DVT) and pulmonary embolism (PE).

We report a case of a patient with an intracardiac thrombus straddling a PFO, which was revealed by echocardiography and was treated successfully with an emergency surgery.

## Case presentation

An obese 55-year-old man (body mass index, 29.4 kg/m^2^) presented at the emergency room of our hospital with dyspnea and cough. He was returning home from a business trip to Kuwait on a 34-h long flight. His symptoms began just after the aircraft landed.

**Figure 1:**
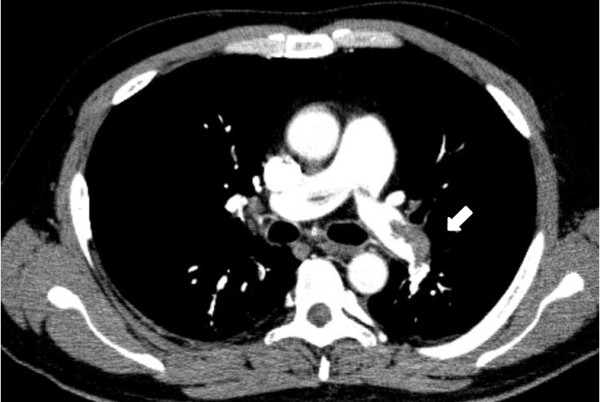
CT scan showing submassive PE.

**Figure 2:**
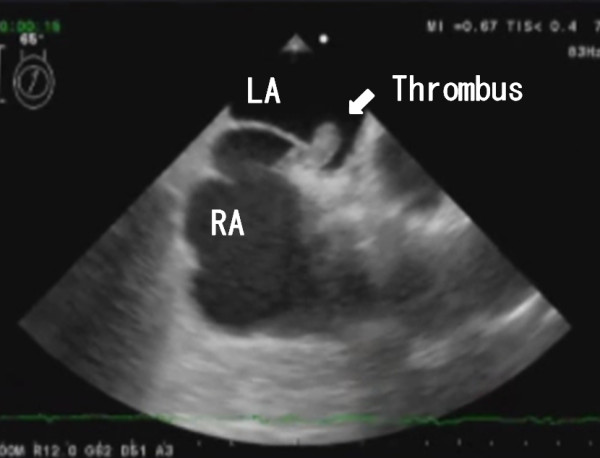
Transesophageal echocardiogram showing a thrombus entrapped in a patent foramen ovale.

His blood pressure was 143/81 mmHg, and blood tests revealed elevated inflammatory markers: white blood cell count was 10200/μl and C-reactive protein level was 8.13 mg/dl. The D-dimer level was 14.6 μl/ml. The patient was desaturated; arterial blood gas analysis at room air revealed O_2_ partial pressure of 44.9 mmHg and O_2_ saturation level of 80%. Other laboratory data were within normal range. Electrocardiography revealed sinus tachycardia (heart rate, 100 beats/min). He did not present with any neurological abnormalities or ischemia of the extremities. Chest X-ray did not reveal any remarkable signs. Computed tomography (CT) demonstrated submassive PE (Figure 1) and DVT. Head CT revealed no remarkable findings. A transthoracic echocardiogram (TTE) revealed a suspected intracardiac thrombus. Left ventricular function was normal but right heart overload was detected (estimated RV systolic pressure >50 mmHg). Transesophageal echocardiogram (TEE) confirmed the presence of a thrombus entrapped in a PFO (Figure 2).

**Figure 3:**
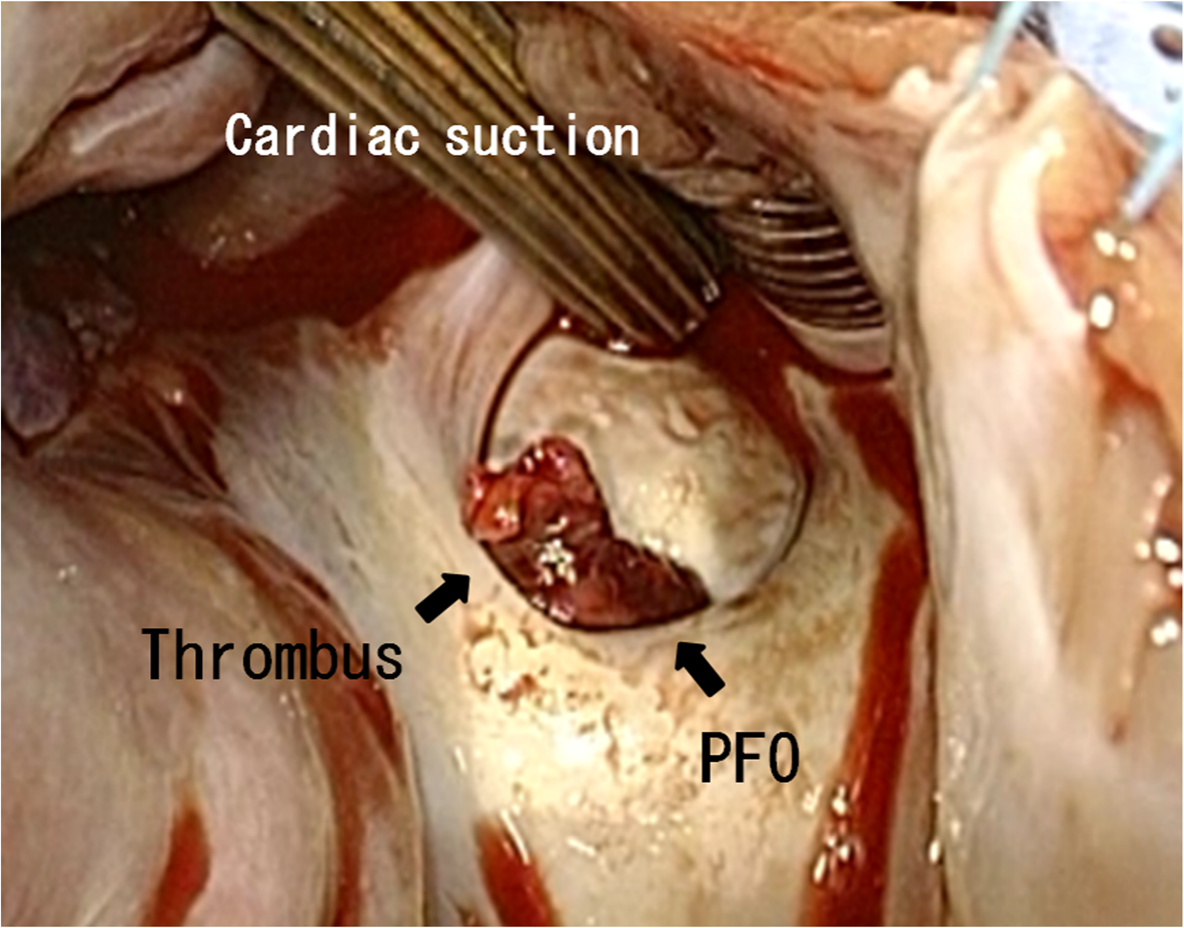
The thrombus viewed from the right atrium.

**Figure 4:**
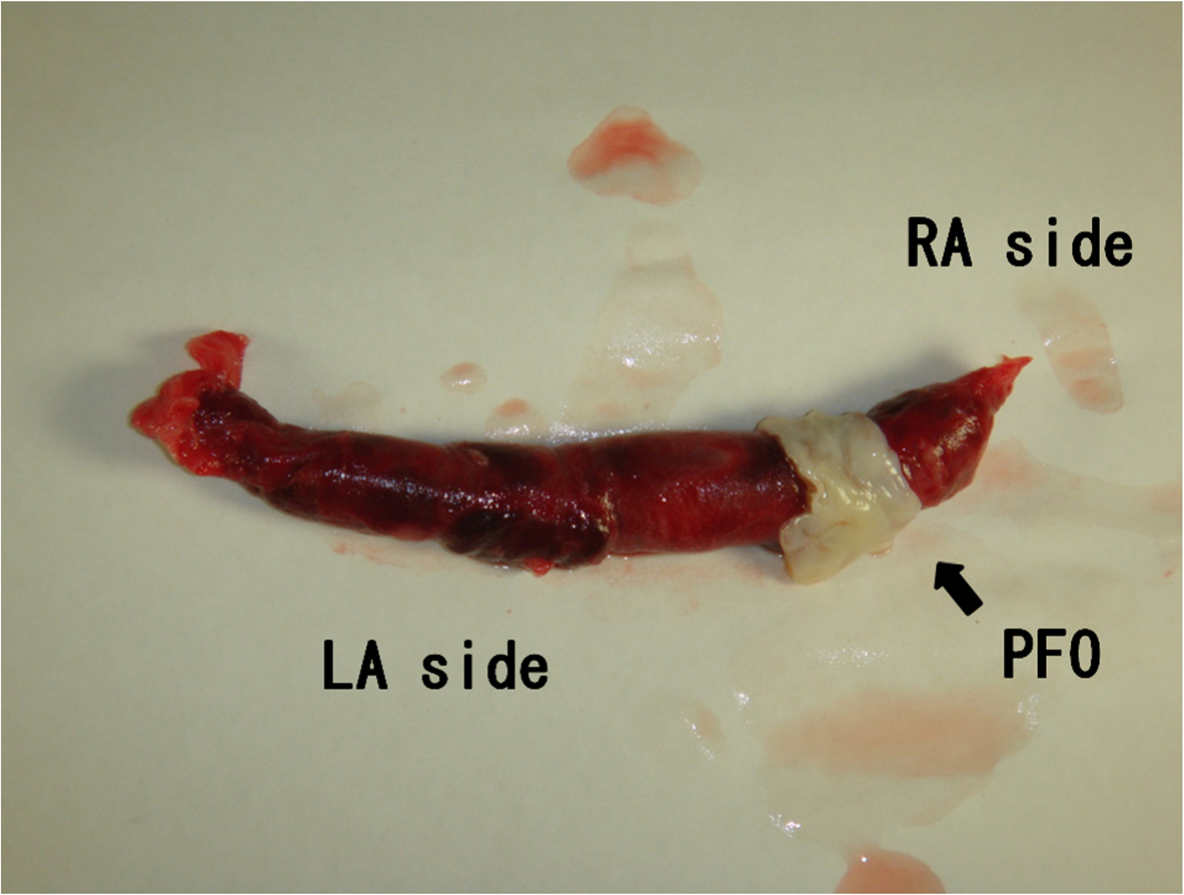
The thrombus with an interatrial septum.

An emergency thrombectomy was performed. Cardiac exposure included a midline sternotomy and a pericardiotomy. Cardiopulmonary bypass was established after aortic and bicaval cannulation with minimal manipulation. Cardiac arrest was achieved with antegrade cold blood cardioplegia, and right atriotomy was performed. A 6-cm long thrombus was removed along with a part of the interatrial septum attached to it (Figures 3 and 4). We removed the thrombus with interatrial septum without touching it so that we prevented the arterial embolism. The septum was closed with fresh autologous pericardium.

We started heparin infusion immediately after achieving hemostasis. And we converted it to Coumadin. The postoperative course was uncomplicated; the patient was discharged and was asymptomatic at the 1-year follow-up.

He underwent bone marrow transplantation for chronic myelogenous leukemia 13 years ago and was under imatinib (Glivec®) therapy for 9 years. His CML status was CR and his platelet count was 24000 /μl at that time. No other remarkable medical history was reported.

## Discussion

PDE occurs after embolic material passes from the venous to the arterial circulation through a right-to-left shunt, frequently a PFO. In The prevalence of a PFO in the normal population is approximately 27% [2], it rarely causes any adverse medical condition. However, during the Valsalva maneuver or after acute elevation in pulmonary arterial pressure due to massive acute PE, a foramen ovale may become patent and result in a catastrophic arterial embolization [3]. A thrombus straddling a PFO in PE is extremely rare and has a high risk of impending PDE (IPDE). The mortality rate with IPDE has been reported to be 16%–21% [3-6]; therefore, IPDE should be promptly diagnosed and treated.

Echocardiography is essential for making a diagnosis [3-8]. The intracardiac thrombus was suspected on TTE; however, TEE was required to confirm the diagnosis. Although TTE is mandatory to detect intracardiac thrombi in patients with DVT or PE, TEE should also be performed, if possible, for detailed observation of the nature of the thrombi and to determine the treatment strategy. A review of previous reports revealed that some patients presented with atypical symptoms; therefore, patients should be carefully evaluated.

Although treatment options include surgery, thrombolysis, and anticoagulation, it is not clear which option is superior and surgery was preferred in several cases.

In the present case, an emergency surgery was performed. Absence of a major cerebrovascular event or systemic emboli before surgery justified the use of cardiopulmonary bypass with systemic heparinization. We decided that pulmonary embolectomy was not necessary because the emboli did not cause hemodynamic instability. No neurological complication was observed after the surgery.

In patients having comorbidities, including advanced age, progressive cancer, stroke, previous cardiac surgery, and hemodynamic instability, thrombolysis and/or anticoagulation therapy are other options [4,7,9]. In our case, the patient walked to the emergency department and presented with dyspnea and cough. He was desaturated but was relatively hemodynamically stable. After oxygen was administered, his symptoms improved and he did not require a ventilator. Therefore, we had ample time to evaluate the patient, and we performed only emergency thrombectomy, which provided good results.

## Conclusion

We should aggressively consider surgical treatment to prevent systemic embolism in this situation.

## Consent

The patient has provided permission to publish these features of his case, and the identity of the patient has been protected.

## Competing interests

The authors report no competing interest and that no funding was received for this study.

## Authors’ contributions

AN wrote the manuscript and collected references. MK and KY helped to revise the manuscript. AN and MK underwent the operation. RY is the chief surgeon and added important comments to the paper. All authors read and approved the final manuscript.
